# *Morinda Officinalis* Polysaccharides Attenuate Varicocele-Induced Spermatogenic Impairment through the Modulation of Angiogenesis and Relative Factors

**DOI:** 10.1155/2019/8453635

**Published:** 2019-04-11

**Authors:** Zhu Zhu, Xiaozhen Zhao, Feng Huang, Feng Wang, Wei Wang

**Affiliations:** ^1^Department of Human Anatomy and Histo-Embryology, School of Basic Medical Sciences, Fujian Medical University, Fuzhou 350122, China; ^2^Department of Pathology, Mengchao Hepatobiliary Hospital of Fujian Medical University, Fuzhou, Fujian, China; ^3^Key Laboratory of Brain Aging and Neurodegenerative Diseases of Fujian Provincial Universities and Colleges, Fuzhou 350122, China; ^4^Research Center for Neurobiology, School of Basic Medical Sciences, Fujian Medical University, Fuzhou 350122, China

## Abstract

Evidence supporting best treatment practices for varicocele is lacking. The effects of a water-soluble polysaccharide extracted from* Morinda officinalis* (MOP) on the progression of varicocele were evaluated in the present study. The extracted MOP was confirmed as having a high purity of 98% with scant protein contamination, and it mainly consisted of glucose, lactose, and xylose at a molar ratio of 7.63:1.23:0.95 glucose:lactose:xylose. MOPs were administered to experimental left varicocele rats immediately after surgery at doses ranging from 25 to 200 mg/kg. As detected by sperm analysis and histopathological staining, the intragastric administration of 100 mg/kg MOPs significantly improved the sperm parameters of bilateral cauda epididymis, attenuated seminiferous epithelial structures, and inhibited germ cell apoptosis. The results of immunofluorescence and immunoblot showed that administration of 100 mg/kg MOPs effectively inhibited angiogenesis in the bilateral testes but modulated the expression of vascular endothelial growth factor (VEGF), matrix metalloproteinase 2 (MMP2), and MMP9 mildly. These results indicate that inhibition of angiogenesis may be one of the mechanisms by which MOP exerts its inhibitive activities on the progression of varicocele, whereas a relative upregulation of VEGF and MMP-9 may be crucial for the spermatogenetic protective effects of 100 mg/kg MOP administration.

## 1. Introduction

Male infertility has been a global social concern because male pathogeny of infertility accounts for approximately 50% of infertility in couples. Varicocele, defined as the dilation and tortuosity of the pampiniform venous plexus in the spermatic cord, is the predominant cause of male infertility due to its prevalence of 45-81% in secondary male infertility and 19-41% in primary male infertility [1]. Varicocele is widely regarded as being responsible for gonadotropin attenuation and spermatogenesis impairment [2]. Dilated and thickened walls of internal spermatic veins, the typical characteristics of varicocele, lead to increased blood stasis and venous volume pressure. Varicocele repair (varicocelectomy) is the common clinical therapy offered to symptomatic or infertile varicocele patients [3–5], and it effectively improves sperm quality while reducing testicle hypoxia and angiogenesis, which are important and adaptive pathophysiological events reported in varicocele patients and in an experimental rat model of varicocele in venous diseases [6, 7]. However, certain defects of varicocelectomy, such as high recurrence rates (7-35%) and controversial effects on pregnancy, make the applications of surgical repair questionable in the era of assisted reproduction [4, 8, 9]. Additionally, the American Society for Reproductive Medicine (ASRM) guidelines recommend against the surgical correcting of subclinical varicocele, which has a prevalence of 55-70% in infertile men [10, 11]. Evidence supporting best treatment practices for varicocele is lacking, and there is an especially low level of evidence to support radiological or surgical intervention for varicoceles in children and adolescents [12]. Thus, the development of effective medicine to prevent the progression of varicocele is urgently needed.

*Morinda officinalis* (*M. officinalis*), of the family Rubiaceae, is a vine that has been widely cultivated in southeastern China for more than 2,000 years and whose roots are frequently added to local soups as a nutrient supplement. The dried roots of* M. officinalis* have long been considered as aphrodisiac tonics for young males, and they have been used in 103 Chinese traditional medicine preparations for the treatment of many diseases, such as impotence, menstrual disorders, depression, osteoporosis, and inflammation [13–16].* M. officinalis* contains carbohydrate constituents, iridoid lactone, anthraquinone, iridoid glucoside, and other compounds [17]. Previous studies have reported that the aqueous extract from* M. officinalis* could significantly increase the sperm count and number of seminiferous cells in rats with impaired reproduction [18, 19]. Water-soluble polysaccharides of MO (MOPs), one of the main bioactive components in this aqueous extract, account for 7-21% of the dry weight of* M. officinalis*, and they have been previously reported to increase the number of seminiferous cells and promote hypothalamic GnRH secretion in varicocele-induced reproductive disorder [20, 21]. However, MOPs are effective in attenuating the sperm count and testicle morphology at a wide range of doses according to the previous studies (50-300 mg/kg body weight), but the optimal effective dose and the definite mechanisms involved in spermatogenic epithelium repair are still unclear.

In the present study, crude MOPs extracted via water extraction and alcohol precipitation were administered to experimental left varicocele (ELV) rats to investigate the effects on varicocele-induced testicular angiogenesis. The results are conducive to assess the optimal dosages and identify the mechanisms of MOPs in varicocele progression.

## 2. Materials and Methods

### 2.1. Extraction, Purification, and Analysis of MOPs

*M. officinalis* dried roots purchased from Fuzhou herb market (Fujian, China) were used to extract water-soluble MOPs by the water extraction and alcohol precipitation method. The crude MOPs were deproteinized by using Sevag reagent [20].

The phenol-sulfuric acid colorimetric method, using glucose as a standard, was used to determine the total carbohydrate content of MOPs [22]. The Bradford method, using bovine serum as a standard, was used to quantify the protein content [23]. The ultraviolet-visible (UV) spectra of the samples were recorded using a UV-2102PC spectrophotometer (UNICO, Shanghai), and the IR spectrum was recorded with a Spectrum 65 FT-IR spectrometer (PerkinElmer, Waltham) in the range of 400-4000 cm^−1^. The homogeneity and molecular weight of the MOPs were evaluated by high-performance gel permeation chromatography (HPGPC) using a dextran standard to calibrate the column and establish a standard curve. After the MOPs were hydrolyzed with trifluoroacetic acid (TFA), the product was analyzed using an Agilent 1260 liquid chromatograph (Agilent Technologies, Santa Clara) with an Alltech 2000 evaporative light scattering detector.

### 2.2. Animals, ELV Model Establishment, and Groups

Seventy mature male Sprague-Dawley rats (6 weeks old, 200 ± 20 g) purchased from the Laboratory Animal Center of Fujian Medical University (No. SCXK(Min)2012-0001) were maintained five to a cage at 22°C on a 12-h light/dark cycle. All animal experiments and procedures were approved and supervised by the local ethics committee (No. 2015-29) and conducted in compliance with the National Research Council's guidelines.

Rats were randomly divided into the following seven groups (n=10 per group): sham-operated group (SO group), ELV group, double-distilled water group (ddH_2_O group), and four MOP-treated groups that were treated with MOP at doses of 25 mg/kg, 50 mg/kg, 100 mg/kg, and 200 mg/kg (M25, M50, M100, and M200 groups, respectively). An ELV rat model was established by partially ligating the left renal vein as previously described [24]. MOPs were dissolved in ddH2O to formulate into solutions with concentrations of 12.5 mg/ml, 25 mg/ml, 50 mg/ml, and 100 mg/ml, which were intragastrically administered to rats in group M25, M50, M100, and M200. The animals were subjected to 6-week medication after reviving from analepsia. The experimental animals were intragastrically administered at 8 am every day, with the dose calculated on the rats' weight of the day. Rats in ddH2O group were intragastrically administrated 2 ml/kg ddH2O instead. After gavage administrated each morning, the rats were sent to metabolic cages (3M12B440, Tecniplast, Varese, Italy) one per cage to collect 24-hour urine samples and stool samples. An animal was excluded if 24-hour urine or stool abnormally reduced during medication. An animal was included in the study only if renal atrophy did not occur.

### 2.3. Sperm Analysis

Sperm suspensions from the bilateral cauda epididymis were stained and analyzed following a method previously described [21]. The sperm count and percentage of rats with sperm morphologic abnormalities were individually observed and recorded by two researchers [25].

### 2.4. Histopathological Staining and Apoptosis Tests

Bilateral testicular tissue samples were harvested, fixed, dehydrated, and made into frozen sections (20 *μ*m) using a cryostat (CM 1950, Leica). Sections were stained with hematoxylin and eosin (H&E) and imaged with an inverted light microscope (Ti-s, Nikon). The Johnsen score (JS) was used to grade testicular injury and spermatogenesis [26]. Seminiferous tubules and interstitial tissue in 10 sections of each specimen were observed, and 5 areas in each section were observed by ×100 magnification to count the number of vessels. The mean number of vessels was recorded as the microvessel density (MVD) of each specimen to evaluate the degree of angiogenesis. Only vessels with a clearly defined lumen or well-defined linear vessel shape were counted as microvessels. Newly formed vessels with only one layer of endothelial cells were excluded.

The* in situ* terminal deoxynucleotidyl transferase-mediated dUTP nick-end labeling (TUNEL) assay was used to detect the apoptosis cells in the seminiferous tubule (*in situ* apoptosis detection kit, ab206386, Abcam). The negative control sections were incubated with dH_2_O instead of TdT labeling reaction mix. Apoptotic germ cells were quantified by counting the number of TUNEL-stained nuclei per seminiferous tubular cross section. Cross sections of 100 tubules per specimen were assessed, and the mean number of apoptotic nuclei per cross section (apoptotic index, AI) was calculated.

### 2.5. Determination of the Expression of Angiogenesis-Related Factors

Frozen sections (20 *μ*m) of bilateral testicular samples were rewarmed, rehydrated, blocked with 10% donkey serum, and incubated with primary antibodies, including antibodies against CD34 (dilution 1:1000, ab81289, Abcam), caspase-3 (dilution 1:100, sc-1225, Santa Cruz), vascular endothelial growth factor (VEGF, dilution 1:500, AB1316, Abcam), p-AKT (dilution 1:1500, #13038, Cell Signaling Technology, Inc.), matrix metalloproteinase 2 (MMP2, dilution 1:250, ab92536, Abcam), and MMP9 (dilution 1:500, ab58803, Abcam), at 4°C for 24 h. The controls were incubated with 10% donkey serum but not primary antibodies. The sections were then incubated with secondary antibodies, including Alexa 488-conjugated donkey anti-goat IgG (A-11055, Invitrogen), and Alexa 555-conjugated donkey anti-rabbit IgG (A-31572, Invitrogen). After the nuclei were dyed by DAPI (62247, Thermo Fisher Scientific Inc.), the sections were imaged and analyzed with a laser scanning confocal microscope (SP8, Leica Microsystems, Inc.).

Bilateral testicle samples were homogenized in RIPA buffer (R0278, Sigma-Aldrich). A total of 30 *μ*g of protein was loaded onto gels and subjected to 12% SDS-PAGE. After the proteins were transferred to membranes, primary antibodies targeting VEGF (dilution 1:1000), p-AKT (dilution 1:2000), AKT (dilution 1:2000, #2920, Cell Signaling Technology, Inc.), MMP2 (dilution 1:1000), MMP9 (dilution 1:1000), and GAPDH (ab 8245, Abcam) were used to detect the protein expressions. After the samples were incubated with horseradish peroxidase-conjugated secondary antibodies (ab6721, ab6789, ab6885, Abcam), the bands were visualized with ECL Western blotting substrate (ab65623, Abcam), and the relative integrated intensities of the bands were expressed as the integrated intensity divided by the intensity of GAPDH.

### 2.6. Statistical Analysis

Image-Pro Plus 6.0 (Media Cybernetics, Inc.) was used to analyze the immunofluorescence and immunoblot pictures, and SPSS 20.0 (IBM) was used to conduct statistical analysis. After tested by the one-sample Kolmogorov-Smirnov test, normally distributed continuous data were expressed as the mean ± standard deviation (SD). Statistical differences were analyzed using one-way analysis of variance (ANOVA), then the least significant difference test was performed to identify differences between two groups. P<0.05 was considered statistically significant.

## 3. Results

### 3.1. Physicochemical and Structural Characterization of MOP

**Figure 1:**
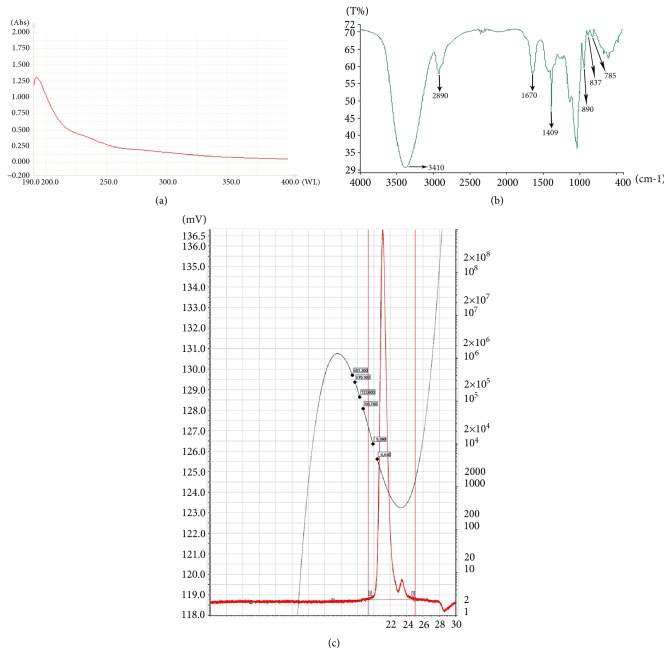
*UV and FT-IR spectra and HPGPC profile of MOP.* (a) UV spectra. (b) FT-IR spectra. (c) A dextran standard with different molecular weights was used to establish a standard curve (black curve). There was one single and symmetrical peak on the HPGPC profile of MOP (red curve).

No characteristic absorption peaks for either proteins or nucleic acids were detected at 280 and 260 nm on the UV spectra (Figure 1(a)). The total carbohydrate content of MOP was 97%, as determined by the phenol-sulfuric acid method. The IR spectrum of MOP showed several absorption peaks related to polysaccharide moieties at 3410, 2890, 1670, 1409, 1258, 1080, 890, 837, and 785 cm^−1^ (Figure 1(b)). There was one single and symmetrical peak on the HPGPC profile of MOP, indicating that the MOP was a homogeneous polysaccharide with a weighted average molecular weight of 1141, a raw average molecular weight of 1648, and a polydispersity of 1.444 (Figure 1(c)). Additionally, MOP comprised six types of monosaccharides: glucose, lactose, xylose, maltose, fructose, and sucrose, and the molar ratio for these was 7.63, 1.23, 0.95, 0.87, 0.72, and 0.64, respectively, which indicated that glucose was the predominant monosaccharide in MOP.

### 3.2. The Effects of MOP on Sexual Performance and Sperm Morphology

Compared to the SO group, the sperm count of bilateral cauda epididymis were significantly decreased in the ELV and ddH_2_O groups (*P*<0.05); furthermore, these groups also had higher bilateral percentages of abnormal sperm (*P*<0.05) (Table 1). Compared to the ELV group, the sperm counts of the M50 and M100 groups were increased, and the percentages of abnormal sperm were decreased (*P*<0.05). Additionally, sperm in bilateral cauda epididymis of the M200 group showed an elevated percentage of abnormality, as well as numerous cell debris and fragments, although the bilateral sperm count of the M200 group was significant increased compared to that of the ELV group (*P*<0.05).

### 3.3. The Effects of MOP on Testicular Morphology, Apoptosis, and Angiogenesis

**Figure 2:**
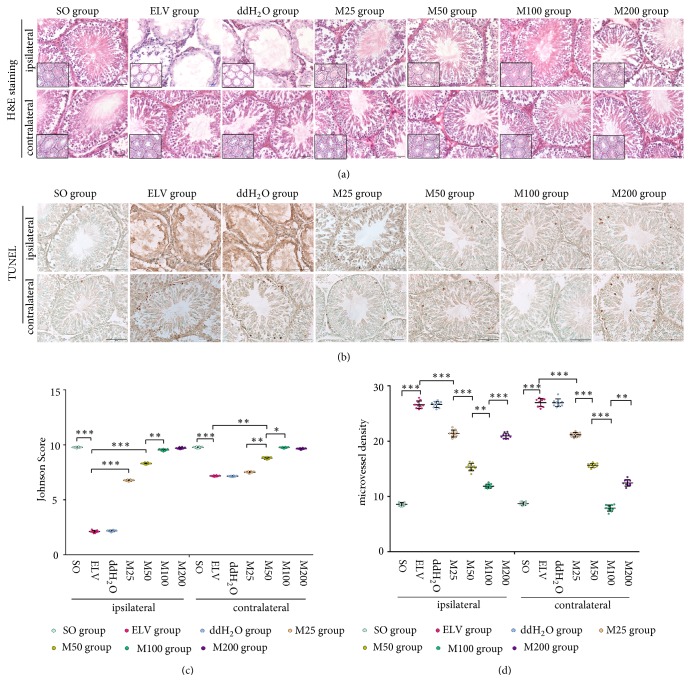
Histopathological staining and apoptosis test results of the experimental groups. (a) H&E staining. (b) TUNEL staining. (c) Johnson scores of bilateral testes. (d) Microvessel density of bilateral testes. *∗*:* P<*0.05; *∗∗*:* P<*0.01; *∗∗∗*:* P<*0.001.

Compared to normal testicular histology and spermatogenesis, the ipsilateral seminiferous tubules of the ELV and ddH_2_O groups showed severe damage, such as the absence of spermatogenic cells and impaired interstitial tissue, and the contralateral seminiferous tubules also showed disordered germinal cell arrangement and increased cellular debris in the lumen. The bilateral spermatocyte count increased after MOP treatment and was accompanied with reduced cell loss, increased degree of ordered cell arrangement and more sperm in the lumen. However, despite the presence of spermatocyte edema and disordered germinal cell arrangement in several seminiferous tubules, the bilateral testicular tissues from rats in the M100 group showed a well-preserved testicular histology (Figure 2(a)).

As shown in Figure 2(c), the JS of bilateral testis in the ELV and ddH_2_O groups was significantly decreased compared to the SO group (*P*<0.05). The JS of bilateral testis in the MOP-treated groups was significantly increased compared to that of the ELV group (*P*<0.05). The JS of bilateral testis in the M50 group was significantly increased compared to that in the M25 group (*P*<0.05), and the JS of bilateral testis in the M100 group was significantly increased compared to that in the M50 group (*P*<0.05). Compared to that in the SO group, the JS of ipsilateral testis in the M200 group showed no significant difference, while the JS of contralateral testis was significantly decreased (*P*<0.05).

The MVD of bilateral testis in the ELV and ddH_2_O groups was significantly increased compared to that of the SO group (Figure 2(d),* P*<0.05), while the MVD of bilateral testis in the MOP-treated groups was significantly decreased compared to that in the ELV group (*P*<0.05). The MVD of bilateral testis was lowest in the MOP100 group among all the MOP-treated groups (*P*<0.05).

As detected by TUNEL staining, several apoptotic cells, including spermatogonial cells and Sertoli cells, were observed in the bilateral seminiferous tubules of the SO group (Figure 2(b)). For the severe damage of lumen cells, it was difficult to calculate apoptotic spermatogenic cells in the ipsilateral testis of the ELV group and ddH_2_O group, and the AI of these groups were marked as ∞. The AI of contralateral testis in ELV group and ddH_2_O group was significantly increased compared to that in the SO group (Table 1,* P*<0.05). The AI of contralateral testis in the MOP-treated groups was significantly decreased compared to that in the ELV group (*P*<0.05). Among all the MOP-treated groups, the bilateral AI in M100 group was lowest, and the bilateral AI in the M200 group was the highest (*P*<0.05, Table 1).

**Figure 3:**
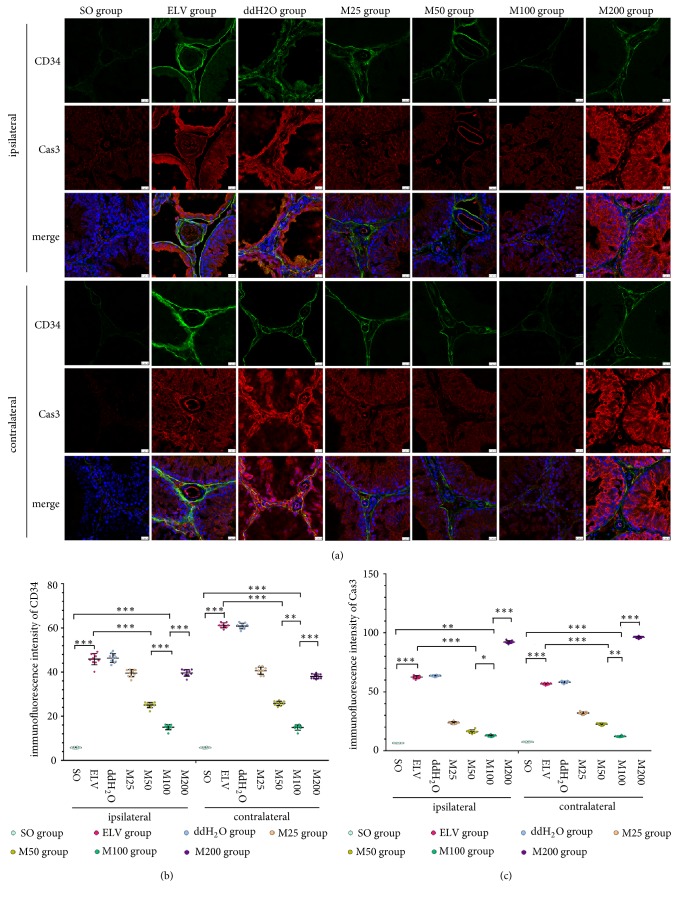
Effects of MOPs on expression of CD34 and Cas3 in bilateral testes. (a) Immunofluorescence of CD34 and Cas3 in bilateral testes. Green: CD34. Red: Cas3. Blue: nucleus. (b) Immunofluorescence intensities of bilateral CD34. (c) Immunofluorescence intensities of bilateral Cas3. *∗*:* P*<0.05; *∗∗*:* P*<0.01; *∗∗∗*:* P*<0.001.

The caspase-3-immunofluorescence-stained sections of the ELV group and ddH_2_O group showed that positive caspase-3 reactions were found in the nuclei and cytoplasm of spermatogenic cells and basement membrane of seminiferous tubules in both the endothelium and adventitial of vessels and cells in the interstitial spaces, as shown in Figure 3(a). Compared to those in the ELV group, the fluorescence intensities of caspase-3 in the bilateral testis of the M25, M50, and M100 groups were significantly decreased (*P*<0.05), while the fluorescence intensities of caspase-3 in the bilateral testis of the M200 group were significantly increased (*P*<0.05). Positive caspase-3 reactions were observed in the nuclei and cytoplasm of spermatogenic cells but not in the vessels and cells in the interstitial spaces in the M100 group, and the M100 group showed the lowest expression of caspase-3 in the bilateral testes among all experimental groups (*P*<0.05).

### 3.4. The Effects of MOP on Angiogenesis-Related Factors

In the ipsilateral testis from the ELV group, immunofluorescence imaging indicated that CD34 immunoreactivity was mainly shown in the basement membrane of seminiferous tubules and adventitial of vessels in the interstitial spaces, and weak immunoreactivity of CD34 was also found in several spermatogenic cells (Figure 3(a)). The contralateral testes of the ELV group showed a similar result. No obvious CD34 immunoreactivity was observed in bilateral testes sections of the SO group. The fluorescence intensities of CD34 in the MOP-treated groups significantly decreased compared to those in the ELV group (*P*<0.05), and the fluorescence intensities of CD34 in the M100 group were the lowest among those of the four MOP-treated groups (*P*<0.05).

**Figure 4:**
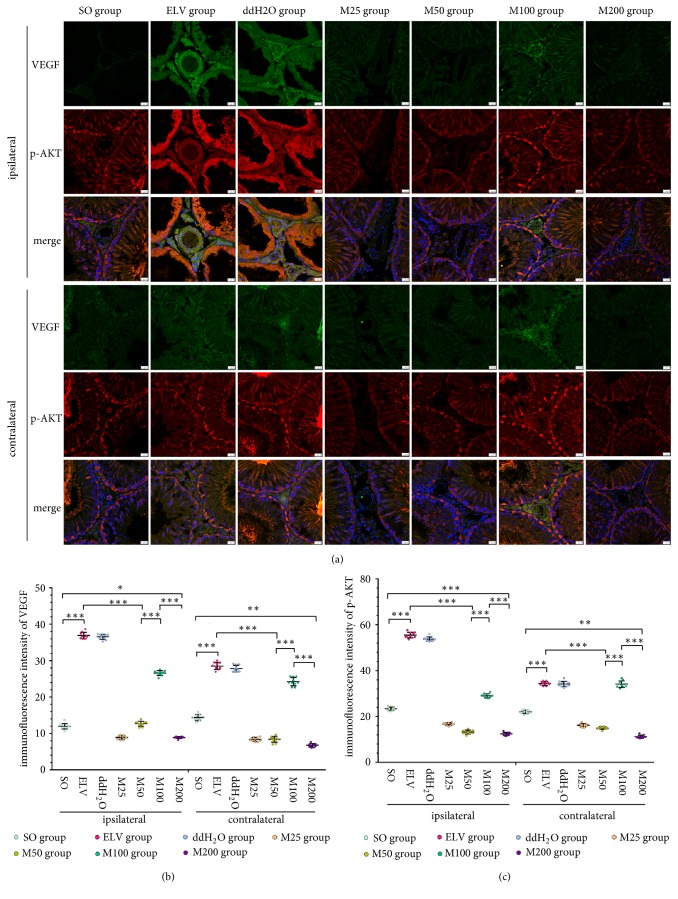
Effects of MOPs on expression of VEGF and p-AKT in bilateral testes. (a) Immunofluorescence of VEGF and p-AKT in bilateral testes. Green: VEGF. Red: p-AKT. Blue: nucleus. (b) Immunofluorescence intensities of bilateral VEGF. (c) Immunofluorescence intensities of bilateral p-AKT. *∗*:* P*<0.05; *∗∗*:* P*<0.01; *∗∗∗*:* P*<0.001.

VEGF immunoreactivity was mainly shown in the seminiferous tubules of several Sertoli cells and in the interstitial spaces of Leydig cells for the bilateral testes from the SO group, whereas VEGF immunoreactivity was also shown in the nuclei of spermatogenic cells and vessels of bilateral testis of the ELV group (Figure 4(a)). Compared to those of the ELV group, the fluorescence intensities of VEGF in the M25, M50, and M200 groups were significantly decreased (*P*<0.05), while no significant difference was shown in the M100 group. For the colocalization of p-AKT and VEGF in testes shown by immunofluorescence, p-AKT immunoreactivity in the bilateral testes of the experimental groups showed similar expression characteristics and trends as those of VEGF.

**Figure 5:**
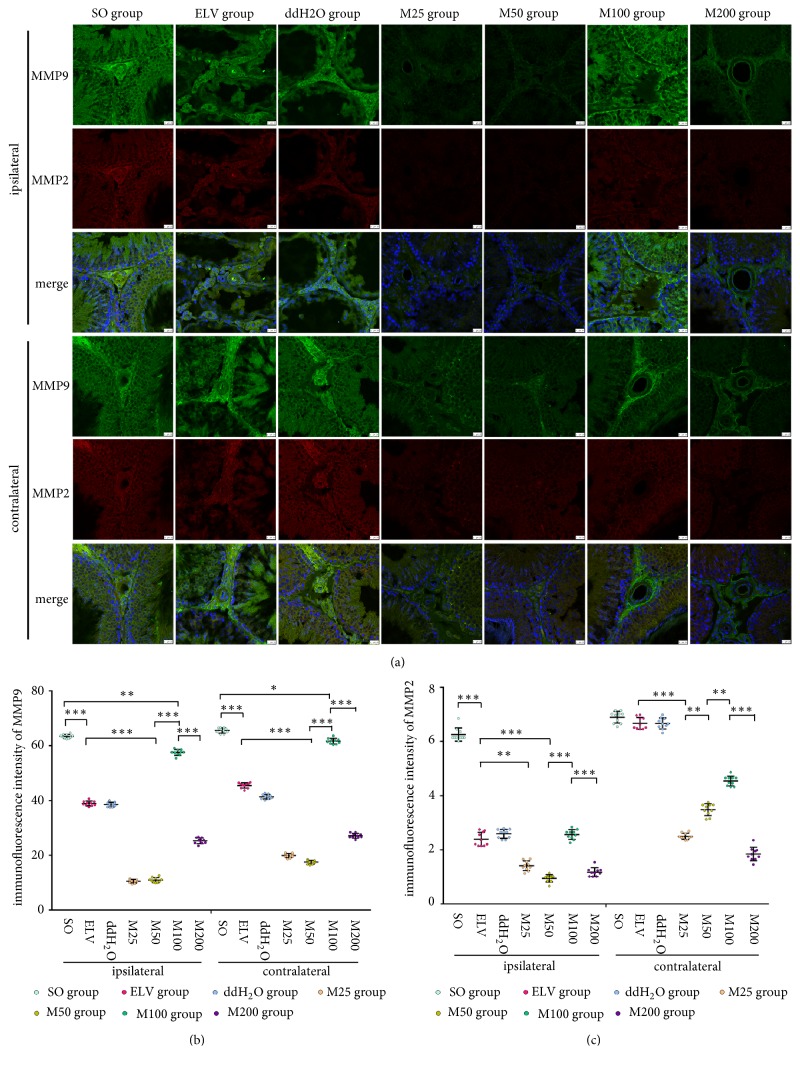
Effects of MOPs on expression of MMP2 and MMP9 in bilateral testes. (a) Immunofluorescence of MMP2 and MMP9 in bilateral testes. Green: MMP9. Red: MMP2. Blue: nucleus. (b) Immunofluorescence intensities of bilateral MMP9. (c) Immunofluorescence intensities of bilateral MMP2. *∗*:* P*<0.05; *∗∗*:* P*<0.01; *∗∗∗*:* P*<0.001.

MMP-2 and MMP-9 immunoreactivity was mainly shown in the nuclei and cytoplasm of spermatogenic cells, Sertoli cells, and Leydig cells, as well as in the vessels of interstitial spaces. Compared to that of the SO group, the fluorescence intensities of MMP-2 and MMP-9 in the ELV group and M100 group showed no significant differences, while the fluorescence intensities of MMP-2 and MMP-9 in the M25, M50, and M200 groups were significantly decreased (Figure 5,* P*<0.05).

**Figure 6:**
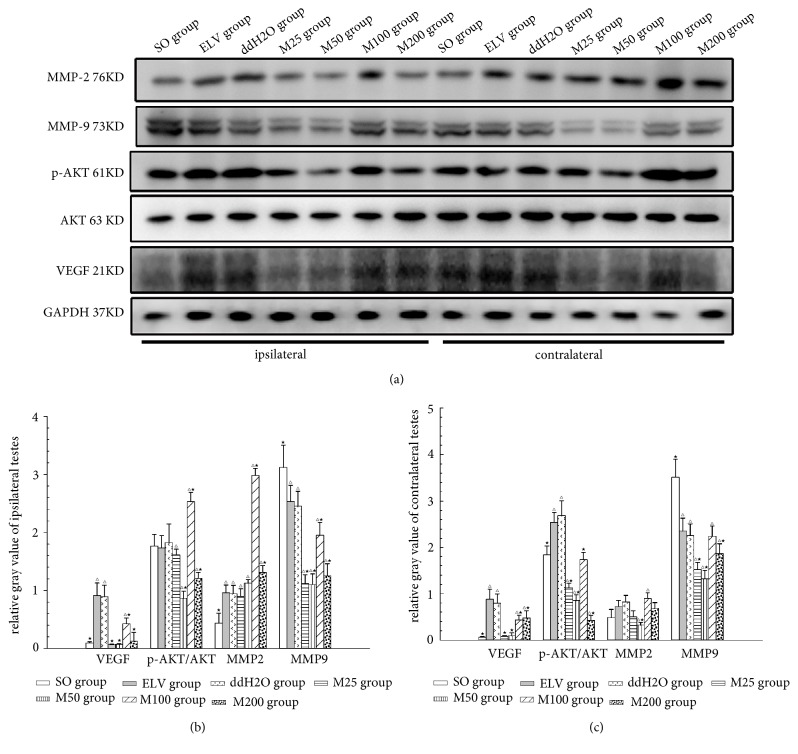
Effects of MOPs on the expression of MMP2 and MMP9 in bilateral testes. (a) Immunofluorescence of MMP2 and MMP9 in bilateral testes. Green: MMP9. Red: MMP2. Blue: nucleus. (b) Immunofluorescence intensities of bilateral MMP9. (c) Immunofluorescence intensities of bilateral MMP2. *∗*:* P*<0.05; *∗∗*:* P*<0.01; *∗∗∗*:* P*<0.001.

The Western blot results showed that, compared to the those in the SO group, the relative intensities of VEGF and the relative ratio of phospho-AKT to the total counterpart were significantly increased in the ELV and ddH_2_O groups compared to the other groups (Figure 6,* P*<0.05). However, the levels of these proteins were significantly decreased in the M50 and M100 groups (*P*<0.05) compared to in the ELV group. The expression of contralateral MMP2 in the ELV group was significantly increased compared to that in the SO group (*P*<0.05), and 100 mg/kg MOP treatment significantly increased both ipsilateral and contralateral MMP2 more than those in the ELV group (*P*<0.05). Both the ipsilateral and contralateral MMP9 of the ELV group were increased compared to those of the SO group (*P*<0.05). MOP treatment significantly decreased contralateral MMP9 compared to ELV (*P*<0.05), while both the ipsilateral and contralateral expression of MMP9 in M100 group were highest among the MOP-treated groups (*P*<0.05).

## 4. Discussion

In this study, a classic experimental varicocele rat model was established, and MOP treatment immediately after surgery was performed, which aimed to illustrate the effects of MOP on the progression of varicocele. The results showed that 100 mg/kg MOP treatment significantly attenuated injury to the seminiferous epithelium and decreased germ cell apoptosis. It is notable that 100 mg/kg MOP showed excellent inhibitive effects on angiogenesis but mild inhibition on some crucial angiogenesis-related factors, such as VEGF, MMP2, and MMP9. Effective angiogenesis inhibition without excessive inhibition of VEGF and MMPs may be one of the major mechanisms and advantages of MOP administered at an early stage of varicocele.

Apoptosis, which occurs during normal spermatogenesis, has a critical regulatory role in spermatogenesis. Increased apoptosis of germ cells has been reported in specimens of male patients with varicocele and in samples from rats with varicocele in the rat model [27, 28]. As detected by TUNEL staining and as evaluated by AI in this study, bilateral testicular apoptosis levels in the ELV group were increased by more than 5 times compared to those in the SO group, which indicated the varicocele rat model was successfully established in this study. MOP, at the dose of 25-100 mg/kg, attenuated bilateral germ cell apoptosis in a dose-dependent manner. Immunofluorescence and immunoblotting of caspase-3, which is the major executioner protease within the apoptotic cascade, showed similar differences among experimental groups on TUNEL staining, which indicated 100 mg/kg was the optimal dose of intragastric-administered MOP to attenuate bilateral testicular apoptosis.

Angiogenesis that refers the growth of new vessels has been reported as a visible response to hypoxia caused by varicocele, which decreases the apoptosis of vascular cells and attributes to dilated and thickened walls of vessels [6]. Angiogenesis, for the progression of varicocele, is a double-edged sword in the progression of varicocele: angiogenesis attenuates increased blood stasis and venous pressure in varicocele, whereas angiogenesis may aggravate the venous volume in ipsilateral testes and slow blood reflux. Although angiogenesis has been generally reported in varicocele, current studies about the effects of angiogenesis on the progression of varicocele are still inconsistent. Polydeoxyribonucleotide has been reported to inhibit the histologic changes in rat experimental varicocele through improving intratesticular VEGF production and vascularization [29]. However, some studies have found that both varicocelectomy and spironolactone administration attenuated spermatogenesis by protecting the tissue from angiogenesis [5, 30]. This study used the microvessel intensity and the expression of CD34 to evaluate angiogenesis, and the results showed 100 mg/kg MOP, which was the most effective dosage in attenuating the bilateral histology of seminiferous epithelium, showed the most significant inhibition of bilateral angiogenesis. Additionally, 25–100 mg/kg MOP showed significant antiangiogenic effects with a dosage-effect relationship, which seems to indicate that MOP attenuates bilateral testicular injury through antiangiogenic effects, while the changes of angiogenesis relative factors were not such consistent with the results of angiogenesis.

VEGF, a specific mitogen of vascular endothelial cells and an angiogenic peptide, has been found to be increased in testicular endothelial cells in varicocele patients and in the germ cell cytoplasm in experimental varicocele rats [31]. In this study, increases of VEGF expression were also detected in germ cells and vessel endothelium. VEGF plays different roles in different cells in the testes: VEGF inhibits the spermatogonial proliferation and spermatogenesis but promotes proliferation and testosterone release of Leydig cells [32, 33]. Administration of 100 mg/kg MOP did not inhibit the contralateral expression of VEGF in the seminiferous tubules, and VEGF expressed in Leydig cells of interstitial tissues increased by more than 10 times. Different from other antiangiogenic drugs or other dosages of MOP, 100 mg/kg MOP showed excellent inhibition of angiogenesis but a specific upregulation on VEGF in the Leydig cells. Phospho-AKT is a crucial downstream protein kinase of VEGF, and its activation has been reported stimulating the expression of both VEGF and MMP-9 [34]. 100 mg/kg MOP administration did not change the expression of phospho-AKT, no matter whether it was in germ cells or in interstitial tissues.

MMPs, the main mediators of extracellular matrix degradation, are involved in pathological processes of connective tissue disease, such as inguinal hernia and chronic venous disorders. MMP-2 and MMP-9 have been found in the interstitium and seminiferous tubules [35], while the changes in MMPs in varicocele are controversial: decreased MMP-2 and MMP-9 levels have been reported in experimental varicocele, while a higher expression of MMP-9 has been reported in varicocele patients. The MMP-9 positive cell ratio did not significantly change in bilateral testes, for both the germ cells and the cells in the interstitium. MOP administration, except the dose of 100 mg/kg, significantly decreased the expression of MMP-9, whereas 100 mg/kg MOP administration significantly increased the MMP-9 level in ipsilateral seminiferous tubule but decreased the ipsilateral interstitium MMP-9 level. The contralateral MMP-9 expression in germ cells and interstitium also has been inhibited. MMP-9 has been reported to serve as the common thread in various diseases in which excessive MMP-9 induces a progressive disorder of collagen metabolism [36]. We speculate that MMP-9 is necessary for the stability of the interstitium, lower expression causes accumulation and widening of the interstitium, and higher expression leads to injury to and loosening of the interstitium. The inconsistencies of the effects of 100 mg/kg MOP on bilateral seminiferous tubules suggest that MMP-9 exerts different effects under different blood stasis conditions. In addition, the effects of MOP on the expression of MMP-2 seem inconsistent with that of MMP-9, which may suggest the different role of these two MMPs in spermatogenesis and interstitium stability.

It is notable that, after one-week intragastric administration of 100 mg/kg MOPs, the faecal output of rats with experimental varicocele increased by 60% compared with that of the ELV group, and the weight gain was 50% lower than that of the ELV group. However, after 2-6 weeks of intragastric administration of 100 mg/kg MOPs, the faecal output and weight gain of rats showed no significant difference with that of the experimental varicocele rats. The above results suggested that MOPs may produce side effects of enhancing gastrointestinal motility and increasing faecal excretion at preliminary stage of administration, lasting for about one week. For MOP with a no observed adverse effect levels value of 100 mg/kg in rats, the human equivalent dose can be estimated by a conversion based on body weight and body surface, which will be 16.2 mg/kg. Thus, the daily dose of MOP for a 60-kg human is about 972 mg. As previous reports, the polysaccharides account for 10-20% of the total weight in* M. Officinalis*, which means the dose of 100 mg/kg MOP is similar to treatment dosage of 4.86–9.72 g* M. Officinalis* which is closed to the treatment dosage 5-10 g in Chinese traditional medicine.

It is worth mentioning that 200 mg/kg MOP significantly increased the apoptosis level of germ cells in contralateral testes detected by TUNEL and caspase-3, and excessive apoptosis may be the reason of high percentage of abnormal sperm in epididymis. The effects of 200 mg/kg MOP administration on inhibition of angiogenesis and upregulation of VEGF and MMP-9 seem equal to 50 mg/kg MOP administration, which reversely validates the effects on angiogenesis and relative factors may be one of the mechanisms of reproductive repair effects of 100 mg/kg MOP.

In this study, the administration period was restricted to 6 weeks to evaluate the apoptosis and angiogenesis in bilateral testes. The observation period was shorter to obtain the long-term prognosis after MOP treatment. Moreover, the timing of medical treatment is important, especially for the adolescent. Although this study indicates the postoperative administration of MOP attenuated varicocele-induced bilateral testicular injuries, the point at which the testicular damage has reached a critical point or the amount of waiting before administrating MOP has not been determined. In addition, the results of this study indicate that the inhibition of angiogenesis without downregulating VEGF and MMP-9 is one of the mechanisms of 100 mg/kg MOP inhibiting varicocele progression, but the underlying reason of this apparent controversy is still unclear. Thus, further studies are required to investigate the changes of some upstream or relative biological events, hypoxia especially.

In conclusion, the MOP extracted and administered in this study had a high purity of 98% and was of high quality. The GPC showed that the polydispersity of the MOP was 1.444 and that the MOP primarily consisted of glucose (retention time, 7.286), lactose (retention time, 13.409), and xylose (retention time, 5.578) at a molar ratio of 7.63:1.23:0.95, respectively. Administration of 100 mg/kg of MOP attenuated the disordered structure of seminiferous epithelium and assist in spermatogenesis. Inhibition of angiogenesis may be one of the mechanisms by which MOP exerts its activities, while relative upregulation of VEGF and MMP-9 may be crucial for the spermatogenetic protective effects of 100 mg/kg MOP administration.

## Figures and Tables

**Table 1:** Sperm parameters and testes apoptosis value of the experimental groups.

	sperm count		abnormal sperm (%)		Testes apoptosis (AI)	
	ipsilateral	contralateral	ipsilateral	contralateral	ipsilateral	contralateral
SO group	196.38±13.64	199.25±9.12	9.83±0.67	9.66±0.84	1.19±0.22	1.18±0.31
ELV group	96.21±9.11^a^	128.63±12.15^a^	53.37±7.89^a^	46.62±6.21^a^	∞^a^	12.87±2.92^a^
ddH_2_O group	98.73±8.99^a^	126.31±9.34 ^a^	51.21±6.36^a^	47.73±7.47^a^	∞^a^	13.36±4.18^a^
M25 group	121.37±14.41^a^	141.63±8.14 ^a^	42.24±3.83^a^	36.63±4.12^a^	7.27±2.18^ab^	7.66±2.38^ab^
M50 group	168.71±11.24^b^	187.71±13.36^b^	21.23±2.47^ab^	16.62±1.34^ab^	5.15±1.18 ^ab^	5.28±1.23^ab^
M100 group	183.43±18.21^b^	194.47±9.22^b^	10.29±0.87^b^	10.08±0.77^b^	3.31±1.12	2.54±1.01^b^
M200 group	162.38±16.88^ab^	183.88±12.37^ab^	48.61±4.29^a^	52.27±5.53^a^	8.36±2.24^ab^	7.87±1.67^ab^

^a^ *P<*0.05 compared to the SO group.

^b^ *P<*0.05 compared to the ELV group.

## Data Availability

The TIF and JPG data used to support the findings of this study are available from the corresponding author upon request. Relevant previously reported data are cited at relevant places within the text as [18–21].
